# Entering the era of precision medicine through multiomics approach

**DOI:** 10.1186/s41232-022-00229-3

**Published:** 2022-09-09

**Authors:** Keishi Fujio

**Affiliations:** Department of Allergy and Rheumatology, Graduate School of Medicine, The University of Tokyo, Tokyo, Japan

Many inflammatory diseases are conditions in which the immune system attacks its own cells. Although diseases are treated with corticosteroids and immunosuppressive drugs in clinics, a variety of treatment problems exist, such as inadequate efficacy or strong immunosuppression, which is complicated by opportunistic infection. However, the recent development of molecular targeted drugs greatly improved the prognosis of inflammatory diseases. For example, nearly half of patients taking molecular targeted drugs achieve clinical efficacy called remission in rheumatoid arthritis (RA) and spondyloarthritis. In retrospect, about half of the cases do not adequately respond to treatment, indicating that the same disease responds differently to specific molecular targets. Similar results were observed in many inflammatory diseases, pointing to the possibility of some differences in the pathogenesis even in cases with the same diagnosis. Therefore, a strategy to predict the response to treatment prior to the start of treatment is now envisioned, which is called precision medicine.

Since a group of patients with RA showed improved prognosis with molecular targeted drugs, parameters that can stratify prognosis have been identified. Clinical parameters such as positive autoantibodies, high inflammatory response, high disease activity, and presence of bone erosions are poor prognostic factors for progressive bone destruction. However, these clinical parameters are present in many RA patients, and it is difficult to achieve high stratification accuracy in individual cases. The key question is whether stratification can be performed using information from the immune cell transcriptome and genome of the peripheral blood and inflammatory sites, which form the basis of the immune response [1].

Both genetic and environmental factors are involved in the development of inflammatory diseases [2]. Many inflammatory diseases are polygenic diseases, and the recently developed polygenic risk score (PRS) enables the evaluation of genetic risk. On the other hand, it has also become clear that environmental factors play a significant role in the severity and poor prognosis of inflammatory diseases. Therefore, highly accurate stratification parameters may be identified by considering the transcriptome and cell ratio of immunocompetent cells, which are influenced by environmental factors. Moreover, combining transcriptome data with genomic information will enable the analysis of immune pathways and immunocompetent cells in relation to genetic predisposition.

In this thematic series review, we invited the leading researchers in the research field of multiomics and single-cell analysis for inflammatory diseases. Dr. Kawashima from Kazusa DNA Research Institute overviewed the current technologies for multiomics analysis, particularly focusing on RNA and protein profiling. Moreover, the importance of bioimaging was demonstrated by the analysis of an autoinflammatory disease, cryopyrin-associated periodic fever syndrome. This autoinflammatory disease is caused by low-frequency somatic mosaicism and cannot be well characterized only by multiomic snapshot analyses of many immune cells.

Dr. Konuma from Osaka University reviewed the recent progress and potential application of PRS. Importantly, PRS could identify a high-risk subgroup of diseases as a predictive biomarker. PRS also provides information on the modifiable risk factors driving prognosis. In the future, PRS may guide drug selection and lifestyle recommendations for various diseases.

Dr. Ota from The University of Tokyo reviewed the expression quantitative trait loci (eQTL)-based analysis of inflammatory diseases. eQTL analysis is useful for the identification of the association between genetic variants and gene expression. By integrating eQTL and GWAS datasets, a transcriptional risk score (TRS) can be developed for patient-level estimation of disease risk. A combination of multi-level information may improve the prediction of these outcomes in inflammatory disease.

Here, we would like to express sincere appreciation to the distinguished researchers in this special issue for sharing their time and expertise. We really hope that these outstanding articles will help readers to understand novel insights into the field of inflammation and regeneration.
